# Optimisation of 3D Printing Parameters and Surface Modification for Porous Gyroid Structures in Beta Titanium Alloy Ti25Nb4Ta8Sn

**DOI:** 10.3390/jfb16110416

**Published:** 2025-11-07

**Authors:** Zdeněk Tolde, Aleš Jíra, Jitřenka Jírů, Vojtěch Hybášek, Vojtěch Smola, Petr Vlčák

**Affiliations:** 1 Department of Physics, Faculty of Mechanical Engineering, Czech Technical University in Prague, Technická 4, 16000 Prague, Czech Republic; vojtech.smola@fs.cvut.cz (V.S.);; 2 Department of Mechanics, Faculty of Civil Engineering, Czech Technical University in Prague, 16629 Prague, Czech Republic; 3 Department of Metals and Corrosion Engineering, University of Chemistry and Technology, Technická 5, 16628 Prague, Czech Republic; jiruj@vscht.cz (J.J.);

**Keywords:** additive manufacturing, Ti25Nb4Ta8Sn, gyroid structures, porous implants, etching

## Abstract

In recent years, 3D printing has become a key technology for producing intricate geometries with high precision. Beta titanium alloys (β-Ti), due to their excellent combination of strength, ductility, low elastic modulus, and biocompatibility, are widely used in the aerospace and medical industries. However, the unique microstructure formed during additive manufacturing characterised by porosity, residual stress, and anisotropy can significantly influence the mechanical performance and durability of these materials. This study examines how different printing parameters affect porosity, dimensional stability, and mechanical properties in the β-Ti alloy Ti25Nb4Ta8Sn. The investigation focuses on thin-walled samples and gyroid structures, which represent model geometries for porous biomedical components. These structures, defined by a periodic network of interconnected channels, provide a useful platform for studying the relationship between geometry and mechanical response. In addition, the effects of surface etching on the morphology and compressive behaviour of printed gyroid structures were evaluated. Compression testing was used to determine how etching alters load-bearing performance and to identify correlations between surface modification and mechanical response. The combined analysis enables optimisation of both printing and post-processing parameters for advanced biomedical applications.

## 1. Introduction

Research in the field of 3D printing of β-Ti alloys for biomedical applications focuses on the influence of printing parameters such as temperature, melting rate, cooling conditions, and post-processing heat treatments on the resulting microstructure and mechanical properties [1,2,3]. In particular, the fabrication of thin-walled samples presents specific challenges, especially due to deformation, thermal stresses, and insufficient substrate adhesion. Thin layers often suffer from excessive cooling, resulting in warping or cracking caused by differences in thermal expansion or rapid temperature gradients.

Selective Laser Melting (SLM) and Laser Powder Bed Fusion (LPBF) are among the most used technologies, as they allow precise control over the microstructure and can significantly enhance mechanical performance, including strength, hardness, ductility, and fatigue resistance [4,5]. For example, higher melting temperatures typically result in a finer grain structure, which translates to better fatigue behavior and mechanical strength.

The optimisation of process parameters is essential, as they directly affect the quality of the printed component. Melting rate influences grain size within the microstructure, which is directly linked to strength and ductility. A slower melting rate often allows more uniform cooling and finer grains, improving mechanical performance, whereas rapid cooling may lead to the development of internal stresses that compromise dimensional stability and long-term performance [6,7,8].

The microstructure formed during the printing process plays a critical role in defining the mechanical characteristics. Several studies have demonstrated that appropriate tuning of the printing parameters enables the development of high strength microstructures without sacrificing ductility [9,10].

An integral component of this optimisation is post processing, particularly chemical etching, which serves multiple roles in surface modification [11]. Etching enables precise adjustment of surface topography and geometric accuracy, alters surface chemical composition and bonding states, and can influence properties such as wettability and fatigue resistance [12,13,14]. Furthermore, controlled etching can assist in stress relief and improve the integration of printed parts in biological environments, making it especially important in biomedical applications.

Additionally, post print heat treatment is essential for the relief of residual stresses and for stabilising the β-phase [15,16], enabling further enhancement of mechanical properties and component lifetime, particularly in demanding applications such as implants or aerospace components.

Porous structures, including gyroids, represent a significant class of architected materials characterised by complex geometries that provide both mechanical robustness and functional advantages. These structures, often derived from mathematical models of triply periodic minimal surfaces, exhibit continuous topology and interconnected porosity [17]. Shapes such as gyroids, Schwarz surfaces, or diamond structures are widely employed in material design where a combination of low density and mechanical strength is required [18,19] such as in biomedical implants (supporting osseointegration and stress distribution), energy systems, aerospace, or filtration.

Additive manufacturing enables precise fabrication of such structures, yet it also introduces several challenges. Key issues include maintaining geometrical fidelity, especially when producing continuous curved surfaces, where limited resolution of the printing system may lead to geometrical deviations. Moreover, microstructural control remains critical due to its direct impact on mechanical performance. Advances in additive manufacturing have enabled the production of a wide variety of architectures, from strut based to shell like morphologies [20,21].

While trabecular designs are suitable for larger porous segments, in small implants the reduced strut diameter may approach the limits of printer resolution, increasing the risk of structural defects [22]. An alternative lies in gyroid based structures, first described by A.H. Schoen [23], whose geometry is mathematically defined by Equation (1):

(1) $$F\left(x,y,z\right)=t=\sin\bar{x}\cdot \cos\bar{y}+\sin\bar{y}\cdot \cos\bar{z}+\sin\bar{z}\cdot \cos\bar{x}$$

where $\bar{x}$ $\bar{y}$ $\bar{z}$ are transformed spatial coordinates and t is a threshold parameter defining boundary curvature and thus the structure’s morphology. By adjusting *t*, the design can be tuned between strut like and shell-like configurations [24].

In metallic materials typically used for printing gyroid structures, thermal gradients during solidification can lead to internal stresses and deformations, adversely affecting strength, toughness, and long-term reliability [25,26].

In addition to β-Ti alloys, which are considered among the most promising materials for biomedical use due to their combination of low elastic modulus, high strength, and excellent biocompatibility, several other alloys and materials are employed for the 3D printing of porous structures. The most widely used system is Ti-6Al-4V, which has become a clinical standard in orthopaedics and dentistry, where it is extensively applied in hip and knee replacements as well as dental implants [27,28]. Cobalt-chromium alloys are commonly used for components requiring high strength and wear resistance, such as dental prostheses and joint replacements. Stainless steels, particularly 316L, are also utilised in implants and surgical instruments owing to their availability and corrosion resistance [29]. A specific category is represented by NiTi shapememory alloys, whose pseudoelastic behaviour and superelasticity make them especially attractive for dynamically loaded implants, including stents and guidewires. In the field of tissue engineering, ceramic materials such as hydroxyapatite and tricalcium phosphate are increasingly used, as they support osteointegration and new bone formation [30,31]; however, their limited mechanical strength restricts their application mainly to smaller or composite implants. A rapidly developing area is also represented by polymeric and composite systems, such as PEEK [32,33], which are frequently combined with bioactive additives to achieve the desired balance of mechanical performance and biological response.

Recent studies have also focused on how pore size and distribution affect both mechanical and biological functionality. While smaller pores often improve strength and density, larger pores enhance mass transport and tissue ingrowth [34]. The ability to customise porous architectures makes them highly adaptable for biomedical and engineering applications [35,36].

Practical biomedical applications of porous structures are primarily concentrated in orthopaedics, dentistry, and vascular surgery [37]. In orthopaedics, porous architectures are employed in large joint replacements, where porosity facilitates osteointegration and ensures more uniform stress distribution between the implant and surrounding bone. In dentistry, porous titanium and cobalt-chromium structures are used for lightweight yet strong dental implants with high levels of biological compatibility [38]. In tissue engineering, porous ceramic or polymeric scaffolds provide suitable matrices for the growth of bone and soft tissue, thereby broadening the possibilities of personalised medicine [39].

Despite the challenges, gyroid and other porous designs remain a subject of intense research due to their outstanding mechanical, transport, and biological properties, rendering them highly suitable for advanced biomedical and engineering systems.

## 2. Material and Methods

### 2.1. Material Preparation and Printing Parameters

**Figure 1:**
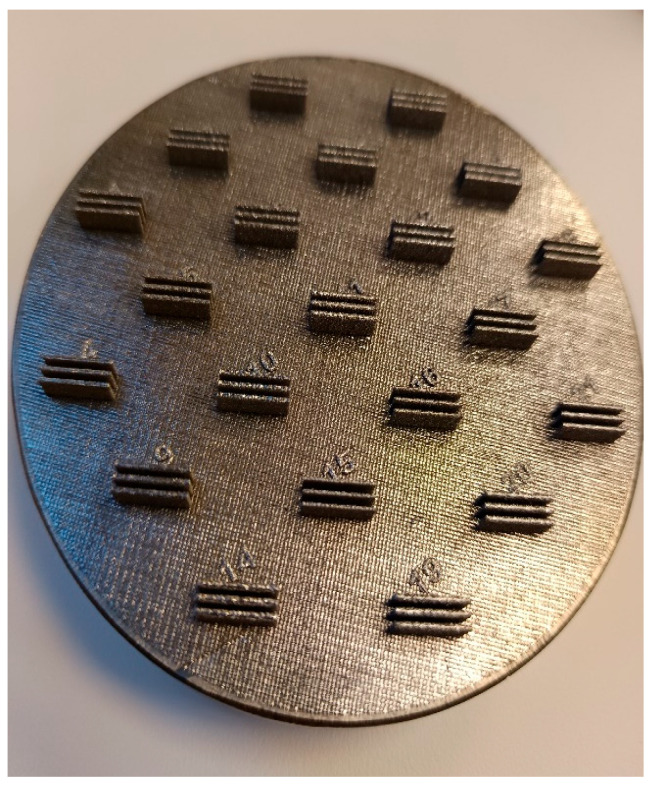
Print reports (samples T1–T21).

For the experimental evaluation of the influence of 3D printing parameters on the quality of the printed samples, a β-titanium alloy Ti25Nb4Ta8Sn (supplied by Advanced Metal Powders s.r.o., Kravaře, Czech Republic) was used, whose chemical composition and properties are described in [40,41]. This alloy was selected primarily due to its stable β-matrix without toxic β-stabilisers and due to its favourable combination of mechanical properties suitable for biomedical use. Niobium, tantalum, and tin function as non-toxic β-stabilisers, enabling retention of the β-phase even after the additive manufacturing (AM) process and the thermal load associated with laser melting. The material exhibits a low elastic modulus (~55–65 GPa depending on the thermal history), which is significantly closer to cortical bone compared with commonly used implant alloys based on α/β titanium systems (for example Ti6Al4V~110 GPa). This helps to reduce the risk of stress shielding in clinical applications. At the same time, sufficient strength and ductility are preserved to ensure structural integrity [42]. The samples were fabricated on a Trumpf TruPrint 1000 (TRUMPF SE + Co., Stuttgart, Baden-Württemberg, Germany) machine at the Institute of Physics of the Czech Academy of Sciences. A total of 21 printing variants (T1–T21) were realized, each comprising three samples with dimensions of 10 × 0.5 mm (see Figure 1).

The baseline printing parameters were: infill laser power 180 W, infill melting rate 550 mm/s, layer thickness 50 μm, number of contours 2, contour laser power 140 W, and contour melting rate 1600 mm s^−1^. The process was carried out in an inert argon atmosphere with a flow rate of 4 L/min, and the oxygen concentration in the chamber did not exceed 0.1%.

The powder material used was analyzed with a Malvern Mastersizer instrument (Malvern Panalytical Ltd., Malvern, UK). The particle size distribution was characterized by values of Dv(10) = 40.9 μm, Dv(50) = 59.3 μm, and Dv(90) = 85.5 μm, with a span of 0.752 indicating a uniform granulometric distribution. The particles exhibited a spherical morphology suitable for the SLM process. Before printing commenced, a standard calibration of the laser system (spot size 55 μm) and a homogeneity check of the power output were performed. The samples were printed in a vertical orientation relative to the build plate to minimize the influence of orientation on dimensional stability and microstructure.

The main variables of the printing setup, summarized in Table 1, included the use/non-use of contour (sample edge), laser movement direction, beam compensation (beam accuracy), and hatch distance (beam width), allowing for systematic investigation of the influence of these parameters on the printing quality of thin samples and the identification of optimal settings to achieve the desired material properties, with potential applications in biomedicine and aerospace.

### 2.2. Gyroid Structure

For the purposes of this study, a series of specimens based on a gyroid structure with a wall like character was prepared. The geometry of the gyroid was generated using MS Lattice software [43]. Each test specimen was designed as a cube with dimensions of 15 × 15 × 15 mm. The specimens were composed of 3 × 3 × 3 elementary gyroid cells, resulting in a total of 27 repeating units arranged in a regular grid.

**Figure 2:**
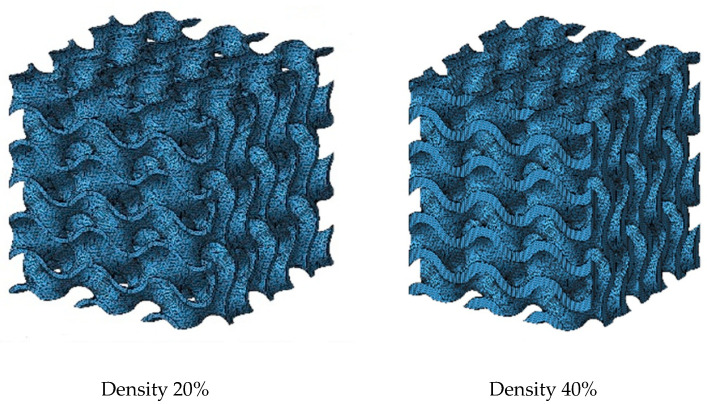
Sample geometries generated in MS Latica and modified in Autodesk Netfabb for 3D printing.

**Figure 3:**
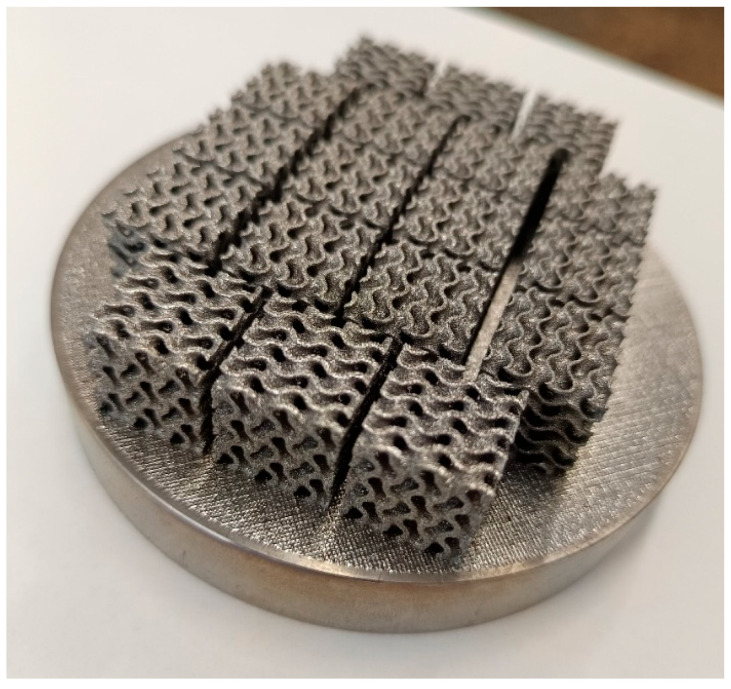
Gyroid 3D printed structure, specimens for compression test.

In this study, the threshold parameter of the gyroid equation was kept constant (*t* = 0) for both variants in order to maintain identical surface topology. The relative densities of 20% and 40% were achieved by modifying the wall thickness, which was defined as an offset along the normal to the gyroid surface. The resulting wall thicknesses were 0.35 mm (20%) and 0.72 mm (40%), respectively. The 20% density variant therefore represented specimens with thinner walls and higher porosity, whereas the 40% variant corresponded to specimens with thicker walls and lower porosity. Both variants were fabricated with identical external dimensions, which enables a direct comparison of the influence of wall thickness and overall density on the mechanical response under compressive loading. The key geometrical parameters of both variants are summarised in Table 2. The specimens were subsequently used for mechanical testing, with their characteristic geometries illustrated in Figure 2 and Figure 3. For each density level, a set of cubic specimens was prepared for compression testing, which enabled not only a comparison of maximum load values and deformation behaviour but also an assessment of experimental repeatability within each series.

### 2.3. Evaluation Methods and Equipment Used

**Figure 4:**
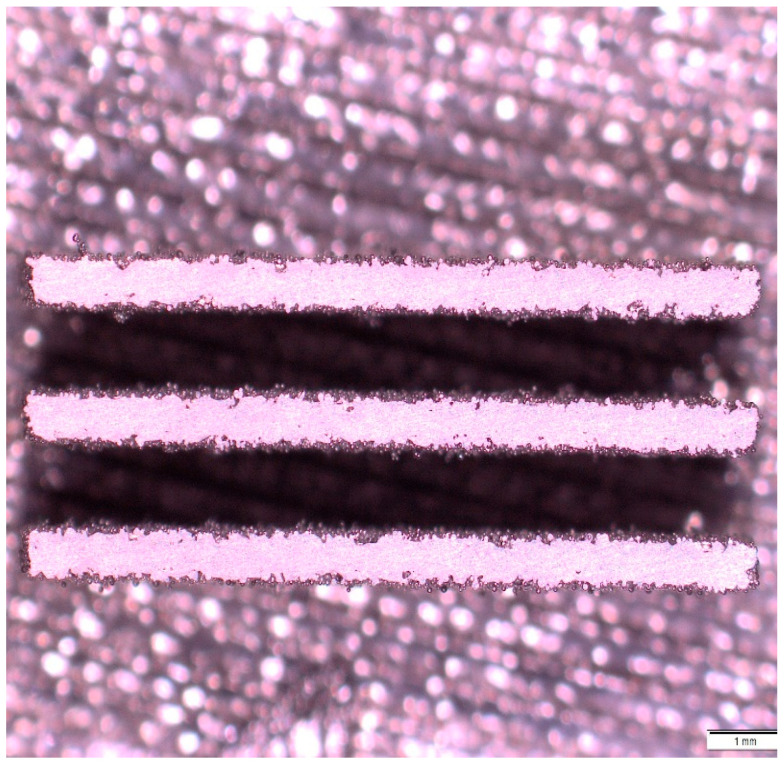
Light microscopy of a T2 sample.

The samples were ground twice (in two levels) on a Bühler (Leinfelden-Echterdingen, Germany) metallographic grinder with abrasive paper grits P240, P320, P640, and P1000. Subsequently, both ground sections were photographed using a metallographic light microscope, as shown in Figure 4 To evaluate the porosity of the 3D-printed structure using image analysis, NIS Elements software (NICON Instruments, Tokyo, Japan) was used. ImageJ software (LOCI, University of Wisconsin, Madison, WI, USA) was used to assess volumetric stability (how much the printed sample area deviates from the specified model). Both measurements were performed on 3 printed samples and in two cutting planes.

**Figure 5:**
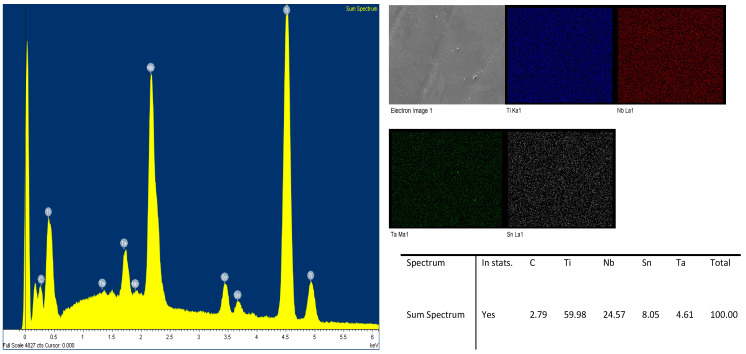
EDS spectrum and elemental maps of the β-Ti25Nb4Ta8Sn alloy surface. The maps confirm a uniform spatial distribution of the elements Ti, Nb, Ta, and Sn. Quantitative results (in wt. %) are as follows: Ti 59.98, Nb 24.57, Sn 8.05 and Ta 4.61.

Samples with a gyroid structure were subjected to compression testing on an Instron device. Two sets of samples (3 each, labeled Sample 1–3) with varying wall thicknesses were loaded at a rate of 0.04 mm per second. For image analysis and verification of the chemical composition were performed using a JEOL JSM 7600-F scanning electron microscope (SEM; JEOL, Tokyo, Japan) equipped with energy-dispersive X-ray spectroscopy (EDS) and utilising secondary electron detectors (SEI, LEI) Figure 5.

### 2.4. Etching as a Post-Printing Surface Refinement Method

To improve the surface quality of the printed gyroid structures, chemical etching was employed, as alternative methods for modifying internal surfaces of such complex geometries are currently not feasible. The primary goal of etching was the removal of residual, partially sintered powder particles, which resulted in altered surface morphology and a reduction in wall thickness.

The etching process was carried out in a Grant XB2 ultrasonic bath. The etching solution was specifically developed for the Ti25Nb4Ta8Sn, β-Ti alloy and consisted of deionised water, nitric acid, and hydrofluoric acid in a volumetric ratio of 5:1:1, with the addition of oxalic acid at a concentration of 8.5 mg mL^−1^. Etching was performed in two cycles of 3 min each.

**Figure 6:**
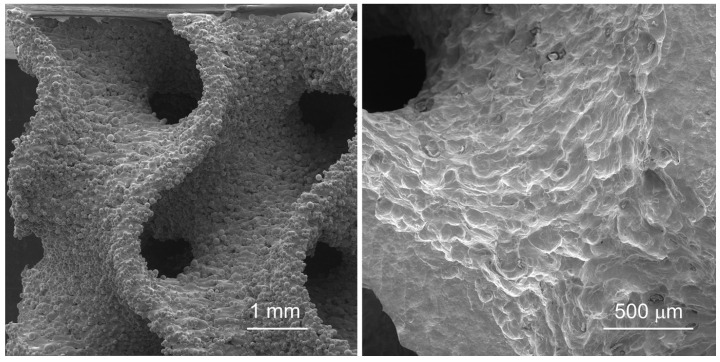
Comparison of the gyroid surface in the as printed state (**left**) and after chemical etching (**right**).

As shown in Figure 6, the left image illustrates the surface morphology of the gyroid structure prior to etching, with numerous adhered powder particles and partially melted agglomerates clearly visible on the surface. These attached particles increase surface roughness and create local heterogeneities, which may serve as potential initiation sites for material failure under cyclic or compressive loading. In contrast, the right image shows the surface after chemical etching, where most of the loosely bound particles have been removed. The etched surface appears smoother, with more clearly defined morphological features and significantly reduced contamination by unmelted powder. This modification not only refines the surface topography but also reduces the effective wall thickness of the structure, thereby influencing both the mechanical response and the biological performance of the material. Measuring the change in wall thickness within the gyroid structure using etching is challenging (due to variable parameters). Therefore, we utilised the weight differential as an indicator of this change. For the gyroid with 20% porosity, the change in weight resulted in a reduction from an initial mass of 3.06 g to 2.42 g, which represents a 21% difference. Conversely, for the gyroid with 40% porosity, the mass was reduced from 7.6 g to 6.51 g, which is a 14.4% difference. The greater the surface area-to-mass ratio, the higher the percentage of material removed by etching.

## 3. Results and Discussion

Following the 3D printing process of the Ti25Nb4Ta8Sn alloy, X-ray diffraction confirmed the presence of a predominantly β-phase with a body centred cubic (bcc) crystal structure. This phase is retained due to the high concentrations of niobium, tantalum, and tin, which suppress the β→α transformation and stabilise the β-phase even under the rapid cooling conditions characteristic of SLM processes. The steep thermal gradients and extremely high cooling rates result in a fine grained, locally textured microstructure without a significant transformation into the martensitic α″ phase. In some regions, minor segregation of tin or niobium may occur along melt pool boundaries, producing slight chemical inhomogeneities; however, these do not lead to any detectable change in phase composition.

After surface etching, no signs of phase transformation or precipitation of secondary phases were detected. Surface analyses indicate that etching selectively affects areas of higher grain boundary energy, where minor oxygen enrichment or local changes in crystallographic orientation may occur. These alterations are confined to the near surface regions and do not compromise the overall stability of the β-matrix. The retention of the β-phase after etching confirms the chemical and microstructural stability of the Ti25Nb4Ta8Sn alloy, which is essential for its long-term performance in biological environments.

### 3.1. Porosity Evaluation

Generally, the porosity in 3D printed products using the SLM method ranges between 0.1–0.4% by volume [44], depending on the setup and printing parameters. Specific porosity requirements can be established depending on the application and material, but these specifications are usually determined by internal standards or customer requirements, rather than a universal norm.

Table 3 summarises the results of the 2D pore area fraction determined from metallographic cross-sections after 3D printing. The values represent the proportion of pores in the analysed area and were evaluated separately after the first and second grinding steps to verify the homogeneity of the structure at different depths of the samples. This metric does not represent volumetric porosity, but a 2D area fraction of pores detected in optical microscopy images. The obtained data clearly confirm that the degree of porosity is strongly influenced by the selected printing parameters, as specified in Table 1. Samples printed with contour (T5–T6) exhibited relatively low porosity, ranging between 0.3–0.5%. However, these samples also showed larger deviations in dimensional stability, which may be related to local overheating and uneven energy distribution caused by multiple laser passes along the contour region.

In contrast, samples printed without contour (T7–T12) generally achieved lower pore area fractions, typically between 0.1% and 0.3%. The best performance was observed for sample T8, where porosity ranged from approximately 0.1–0.2%. This improved homogeneity can be attributed to a more uniform temperature distribution and reduced transition effects between the contour and infill regions. An exception was sample T12, where the use of the “Out to In” strategy resulted in less uniform melting and increased porosity of around 1%.

The next group of samples, T13–T16, focused on the influence of beam compensation. These specimens showed a slight improvement compared with the standard parameters, although the results were not fully consistent. A smaller compensation value (0.001 mm) helped to maintain porosity below 0.5%, whereas a larger compensation (0.12 mm) led to a mild increase in porosity due to local over melting and uneven overlap of adjacent scan tracks.

The highest variability was observed in samples T18–T21, where the combination of modified beam compensation and the “Out to In” strategy resulted in inconsistent behaviour in some cases, porosity decreased below 0.1%, while in others it exceeded 1.7%. This variability can be attributed to differences in local heat accumulation and changes in the melt pool dynamics during laser scanning transitions.

Overall, it can be concluded that the “no contour” strategy tends to yield better results in terms of minimising porosity. However, to achieve reproducible and mechanically stable properties, it is essential to simultaneously optimise beam compensation and scanning strategy, as their mutual interaction has a decisive impact on energy distribution in the melt zone and, consequently, on the overall material quality

### 3.2. Evaluation of the Area of a 3D Printed Sample

The assessment of the prescribed area serves as an indicator of the percentage deviation between the designed dimension of the printed specimen and its actual dimension after printing. Table 4 demonstrates how the selected printing strategies influence this 2D deviation from the nominal CAD geometry. This parameter does not represent true 3D volumetric change, but a 2D area-based deviation determined from microscopic images using ImageJ. No 3D plugins or 3D functionality of ImageJ were used; the analysis was performed exclusively on 2D image data.

The optimal value is the one that most closely approaches 100%. Values lower than 100% indicate undersized specimens relative to the design, whereas higher values reflect excessive melting and material overextension.

The results presented in Table 3 show that samples T1–T6, printed with a contour, exhibit values in the range of 82.9–119.7%, indicating considerable variability in dimensional stability. Most of these samples display a systematic under sizing relative to the design (e.g., T3 = 82.9%, T2 = 87.6%, T4 = 91.3%), whereas sample T6 (119.7%) shows clear over melting and dimensional expansion beyond the designed boundaries. This difference can be explained by the fact that the presence of a contour increases local heat accumulation near the edges of the model, leading to melt pool broadening and subsequent geometric distortion. In addition, multiple remelting passes may cause excessive shrinkage upon cooling when heat transfer is non-uniform.

Samples T7–T12, printed without contour, generally achieved better performance, with values between 95.2–111.9%. The highest dimensional accuracy was observed for sample T7 (98.0%), which closely matched the intended geometry. The absence of a contour helps to reduce local thermal gradients, thereby preventing overheating at the edges and ensuring more uniform heat distribution during the melting process. Conversely, samples T9 (110.4%), T10 (105.3%), and T11 (111.9%) show dimensional expansion caused by a higher effective energy input and the “Out to In” scanning strategy, which concentrates heat toward the centre of the sample and leads to over melting of the internal regions.

In the case of samples T13–T16, where beam compensation was investigated, the measured values range from 91.5–109.8%. Beam compensation modifies the effective laser path, thereby altering the width of the melted zone. Insufficient compensation can result in incomplete melting of the borders, whereas excessive compensation may produce overlapping tracks and overheating. Sample T14 (102.1%) represents an optimal balance between these effects, while T15 (59.1%) and T16 (66.9%) show evidence of significant distortion and melt pool overflow, likely caused by inadequate beam-width correction.

The group of samples T17–T21, which examined the influence of hatch spacing, shows values between 81.9–89.7%. Increasing hatch distance leads to insufficient overlap between melt tracks, which results in local shrinkage and poor inter-track bonding. Conversely, overly small hatch spacing may cause excessive heat accumulation, local expansion of the melt pool, and deformation. These effects directly account for the high variability observed among these samples.

Overall, the best dimensional accuracy was achieved for samples T7 (98.0%) and T14 (102.1%), which combine balanced thermal distribution with optimal beam compensation. The poorest outcome was found in sample T15 (59.1%), indicating severe over melting and thermal distortion, while samples T6 (119.7%) and T11 (111.9%) exhibited the most extensive material expansion.

These findings confirm that dimensional accuracy is governed by a combination of thermal management, melt-track overlap, and scanning strategy. Even minor variations in these parameters can lead to substantial geometric deviations, highlighting the necessity of precise machine calibration and validation of scanning parameters for each specific β-Ti alloy system.

### 3.3. Pressure Test

Compression tests of gyroid structures are used to experimentally characterise their mechanical behaviour under load. In these tests, the specimens are subjected to controlled compression, and the relationship between the applied force and the resulting deformation is recorded up to the point of failure. From these data, parameters such as maximum load capacity, behaviour within the elastic and plastic regions, and the mode of progressive collapse of the porous architecture can be determined.

The results make it possible to compare the influence of geometrical parameters, particularly network density, wall thickness, and pore size or shape, on the mechanical response of the structure. Testing variants with different densities provides information on how load bearing capacity and deformation behaviour change with modifications of gyroid geometry.

**Figure 7:**
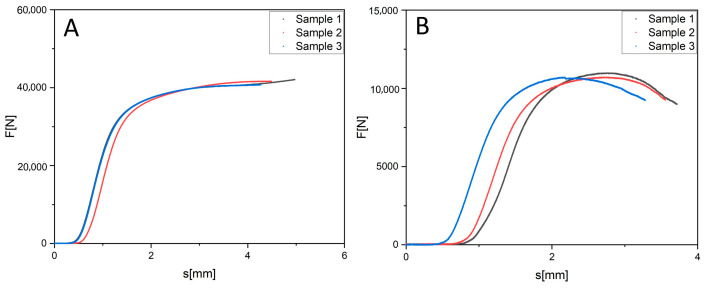
Courses of such a gyroid cube test with 40% density (**A**) and 20% density (**B**).

**Figure 8:**
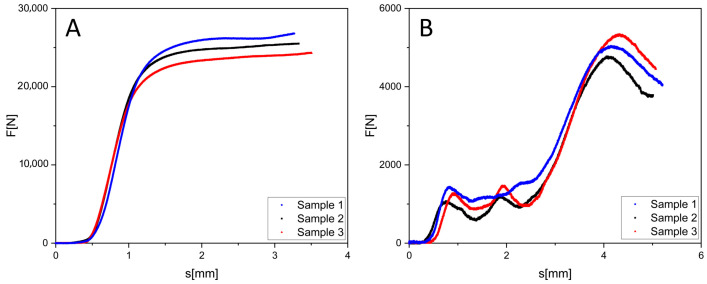
Course of such a gyroid cube test with an etched surface with 40% density (**A**) and 20% density (**B**).

The test series also included specimens subjected to chemical etching. This process removes residual sintered powder particles from the surface and alters the morphology of the walls, which may affect the thickness of the load bearing elements as well as the surface homogeneity. Comparing etched and unetched specimens therefore allows assessment of how surface treatment influences the course of the loading curves and the overall mechanical response of the structures. Figure 7 illustrates the results of the compressive tests conducted on gyroid samples of two different relative densities, 40% and 20%. For the samples with a density of 40% (Figure 7A), the peak force achieved was approximately 45,000 N. The individual load displacement curves exhibit a high degree of similarity (the samples labelled 1–3 are three identically prepared gyroid cubes, as in Figure 8), which confirms the good repeatability of the measurements. The curve profiles display a characteristic non-linear shape: an initial elastic region is followed by plastic deformation, and then by a gradual collapse of the cellular architecture. In the region following the maximum, a relatively stable plateau phase is evident, which corresponds to the progressive failure of individual cells while maintaining the overall structural integrity of the sample.

The samples with a density of 20% (Figure 7B) reached a peak load bearing capacity of approximately 12,000 N, a value significantly lower than that of the 40% density variant. The overall shape of the load displacement curves remained similar; however, following the attainment of the peak force, more numerous irregularities and local force drops appeared. These deviations indicate an uneven failure mechanism, where the thinner walls and higher porosity lead to a less stable collapse. Nevertheless, all three characteristic phases the elastic, plastic, and collapse regions can still be distinguished.

Overall, it can be concluded that a higher relative density of the gyroid structure (40%) results in a substantially greater mechanical load bearing capacity and a more stable deformation progression, whereas the lower density (20%) exhibits lower maximum force values and a more complex failure mechanism.

Figure 8 presents the results of the compressive tests performed on etched gyroid samples with relative densities of 40% and 20%. For the 40% density samples (Figure 8A), the maximum load bearing capacity achieved was approximately 27,000 N, which is almost 40% less than that of the non-etched variant (Figure 7A). The curve profile was maintained the elastic region was followed by plastic deformation and a gradual structural collapse; however, the peak load capacity was substantially reduced. Nonetheless, the individual curves retained a similar trajectory, confirming the reproducibility of the results even following the chemical surface treatment.

For the etched samples with a density of 20% (Figure 8B), the maximum force reached was around 5500 N, less than half the value measured for the non-etched variant (Figure 7B). The deformation progression was accompanied by irregularities in the region following the maximum, which is associated with the wall weakening due to etching and local surface defects. An interesting phenomenon was also observed during the initial loading phase, where the peripheral sections of the sample were compromised the thin outer walls were significantly attenuated by the etching process and began to fail sooner than the central cells.

A comparison of the results from Figure 7 and Figure 8 indicates that while chemical etching effectively removes surface impurities and adhering powder, it simultaneously leads to a significant decrease in the mechanical resistance of the structures. While the reduction in load bearing capacity is substantial for the higher density samples (40%), still leaving the sample with a certain reserve, the lower density samples (20%) experience a dramatic reduction in maximum load bearing capacity to less than half of the original value. This confirms that etching does not act merely as a surface treatment but fundamentally affects the effective wall thickness and, consequently, the overall stability of the load bearing structure.

It is therefore necessary to consider that when designing porous structures intended for applications in loaded environments, etching cannot be viewed solely as a final cleaning step, but as a process that alters the fundamental mechanical properties of the entire component. In practice, this means it is advisable to account for an etching allowance, i.e., an increase in wall thickness during the geometric design, to achieve the required strength level after the chemical wall thinning. This approach allows the benefits of a clean and homogeneous surface after etching to be utilised without unacceptably compromising mechanical integrity.

Concurrently, it must be considered that the negative impact of etching on mechanical properties may vary depending on the specific setting of the printing process, the chemical composition of the alloy, and the etching conditions. Precise determination of the appropriate combination of these factors is therefore essential for optimising the design and manufacture of porous titanium structures intended for medical applications.

## 4. Conclusions

Based on a comprehensive analysis of the dimensional stability and porosity of 3D printed samples of the β-Ti alloy Ti25Nb4Ta8Sn, in relation to detailed printing parameters, it is possible to identify the most suitable processing conditions. Sample T14, with a beam compensation setting of 0.001 mm, demonstrated the most balanced results, achieving dimensional stability values close to 100% and low porosity. These findings indicate that satisfactory outcomes can be obtained through optimised beam compensation, even in the absence of a contour. In contrast, other sample sets exhibited deviations in dimensional stability or increased porosity, confirming the strong dependence of print quality on the interplay between contour, beam compensation, and hatch distance.

Compression tests of gyroid structures further emphasised the importance of these parameters in defining mechanical performance. While non etched structures with 40% and 20% density achieved maximum loads of approximately 45,000 N and 12,000 N respectively, etched structures showed a substantial decrease in load bearing capacity, with values reduced to roughly 27,000 N and 5500 N. Despite similar deformation patterns, the reduction in strength can be attributed to wall thinning and surface defects induced by the etching process.

In conclusion, achieving optimal properties of Ti25Nb4Ta8Sn structures requires not only careful adjustment of beam compensation, hatch distance, and contour use, but also consideration of post processing treatments. Although etching enhances surface activity and may improve biological responses such as osteointegration, it simultaneously reduces mechanical integrity. For this reason, the final application of these structures should be carefully matched to the balance between biological and mechanical requirements.

## Figures and Tables

**Table 1:** Used Print Reports and Parameters.

Sample ID	Modification Type	Description of Printing Parameters/Strategy
T1	Reference sample	Default parameters: laser power 180 W, melting rate 550 mm/s (based on previous optimisation).
T2	Scanning strategy	Changed to chessboard scanning.
T3	Scanning strategy	Offset filling “In → Out” (from centre to edge).
T4	Scanning strategy	Offset filling “Out → In” (from edge to centre).
T5	Scanning strategy	Stripe filling (“Zigzag—Connected”).
T6	Border parameters	Melting rate for border paths adjusted to 600 mm/s and laser power at 180 W.
T7	Contour removal	No contour; border distance = 0 mm; other parameters as T1.
T8	Contour removal	No contour; border distance = 0 mm; other parameters as T2.
T9	Contour removal	No contour; border distance = 0 mm; other parameters as T3.
T10	Contour removal	No contour; border distance = 0 mm; other parameters as T4.
T11	Contour removal	No contour; border distance = 0 mm; other parameters as T5.
T12	Contour removal	No contour; border distance = 0 mm; other parameters as T6.
T13	Beam compensation	Beam compensation = 0.001 mm; other parameters as T1.
T14	Beam compensation	Beam compensation = 0.001 mm; other parameters as T7.
T15	Beam compensation	Beam compensation = 0.12 mm; other parameters as T1.
T16	Beam compensation	Beam compensation = 0.12 mm; other parameters as T7.
T17	Hatch distance	Hatch distance = 0.08 mm; other parameters as T1.
T18	Hatch distance	Hatch distance = 0.12 mm; other parameters as T1.
T19	Hatch distance	Hatch distance = 0.06 mm; other parameters as T7.
T20	Hatch distance	Hatch distance = 0.08 mm; other parameters as T7.
T21	Hatch distance	Hatch distance = 0.12 mm; other parameters as T7.

**Table 2:** Specifications of 3D printed Ti25Nb4Ta8Sn alloy samples for single compression test.

Density	Size [mm]	Cells	Wall Thickness [mm]
20%	15 × 15 × 15	3 × 3 × 3	0.35
40%			0.72

**Table 3:** Percentage of pore area determined from image analysis.

	Sample (Percentage of Pores)																				
	1	2	3	4	5	6	7	8	9	10	11	12	13	14	15	16	17	18	19	20	21
	first grinding																				
**1**	1.8	1.4	4.3	1.1	1.6	0.1	0.7	0.3	0.9	15.9	0.4	0.2	1.2	0.1	1.6	0.2	0.4	2	0.2	0	0.2
**2**	0.6	0.7	2.9	0.2	1.5	0.1	0.3	0	0.7	12.9	0.6	0.1	2.1	0.2	1	0.1	0.3	2.1	0.2	0.3	0.2
**3**	0.4	0.4	0.3	0.2	0.8	0	0.2	0	0.1	7.9	0.5	0.1	1.7	0.2	0.9	0.1	0.4	0.8	0.2	0	0.3
**A** **verage**	0.9	0.8	2.5	0.5	1.3	0.1	0.4	0.1	0.6	12.2	0.5	0.1	1.7	0.2	1.2	0.1	0.4	1.6	0.2	0.1	0.2
**SD**	±0.62	±0.42	±1.66	±0.42	±0.36	±0.05	±0.22	±0.14	±0.34	±3.30	±0.08	±0.05	±0.37	±0.05	±0.31	±0.05	±0.05	±0.59	±0.00	±0.14	±0.05
**1**	0.3	0.6	0.3	0.1	0.3	0.1	0.1	0.3	0.2	1.5	0.1	0.2	1	0.3	1	0.1	0.1	1.1	0.1	0	0.1
**2**	0.7	0.9	0.9	0.3	0.7	0.1	0.1	0	0.1	2.5	0.1	0.3	1.5	0.3	0.8	0.1	0.5	2.1	0	0	0.3
**3**	1	0.5	0.6	0.7	0.9	0.1	0	0.4	0.1	1.4	0.1	0.2	1.2	0.4	1.8	0.1	0.4	2.3	0.1	0.1	0.1
**A** **verage**	0.7	0.7	0.6	0.4	0.6	0.1	0.1	0.2	0.1	1.8	0.1	0.2	1.2	0.3	1.2	0.1	0.3	1.8	0.1	0	0.2
**SD**	±0.29	±0.17	±0.24	±0.25	±0.25	±0.00	±0.05	±0.17	±0.05	±0.50	±0.00	±0.05	±0.21	±0.05	±0.43	±0.00	±0.17	±0.52	±0.05	±0.05	±0.09
**O** **verall** **A** **verage**	0.80	0.75	1.55	0.43	0.97	0.08	0.23	0.17	0.35	7.02	0.30	0.18	1.45	0.25	1.18	0.12	0.35	1.73	0.13	0.07	0.20
**SD**	±0.1	±0.05	±0.95	±0.05	±0.35	±0	±0.15	±0.05	±0.25	±5.2	±0.2	±0.05	±0.25	±0.05	±0	±0	±0.05	±0.1	±0.05	±0.05	±0

**Table 4:** Percentage of compliance with the specified dimensional stability of the sample.

Sample																					
	T1	T2	T3	T4	T5	T6	T7	T8	T9	T10	T11	T12	T13	T14	T15	T16	T17	T18	T19	T20	T21
	First grinding—area after printing (image size 2560 × 1920 px) defined sample size is 182,667 px																				
**px**	176,268	158,677	146,192	169,162	166,902	212,628	177,437	171,482	197,589	190,472	201,802	167,009	197,443	167,134	105,948	124,022	152,936	158,706	148110	165,789	164,297
**Percentage Representation (%)**	96.5	86.9	80	92.6	91.4	116.4	97.1	93.9	108.2	104.3	110.5	91.4	108.1	91.5	58	67.9	83.7	86.9	81.1	90.8	89.9
	Second grinding area after printing (image size 2560 × 1920 px) defined sample size is 286,785 px																				
**px**	258,586	253,528	245,797	258,300	265,992	352,570	283,770	278,352	323,231	304,861	324,914	284,108	320,053	322,965	172,380	188,896	243,935	248,251	237,097	237,329	256,766
**Percentage Representation** **(%)**	90.2	88.4	85.7	90.1	92.7	122.9	98.9	97.1	112.7	106.3	113.3	99.1	111.6	112.6	60.1	65.9	85.1	86.6	82.7	82.8	89.5
**Overall Average (%)**	93.3	87.6	82.9	91.3	92.1	119.7	98	95.5	110.4	105.3	111.9	95.2	109.8	102.1	59.1	66.9	84.4	86.7	81.9	86.8	89.7
**SD**	±3.15	±0.75	±2.85	±1.25	±0.65	±3.25	±0.9	±1.6	±2.25	±1	±1.4	±3.85	±1.75	±10.55	±1.05	±1	±0.7	±0.15	±0.8	±4	±0.2

## Data Availability

The original contributions presented in this study are included in the article. Further inquiries can be directed to the corresponding author.
